# Rhynchophylline Attenuates Senescence of Endothelial Progenitor Cells by Enhancing Autophagy

**DOI:** 10.3389/fphar.2019.01617

**Published:** 2020-01-28

**Authors:** Lin Lin, Lei Zhang, Xin-tong Li, Jing-kang Ji, Xiao-qing Chen, Yun-lun Li, Chao Li

**Affiliations:** ^1^ Institute of Traditional Chinese Medicine Innovation, Shandong University of Traditional Chinese Medicine, Jinan, China; ^2^ The First Faculty of Clinical Medicine, Shandong University of Traditional Chinese Medicine, Jinan, China; ^3^ Institute of Education and Psychological Sciences, University of Jinan, Jinan, China; ^4^ Faculty of Traditional Chinese Medicine, Shandong University of Traditional Chinese Medicine, Jinan, China; ^5^ Experiment Center, Shandong University of Traditional Chinese Medicine, Jinan, China; ^6^ Department of Cardiovascular, Affiliated Hospital of Shandong University of Traditional Chinese Medicine, Jinan, China

**Keywords:** endothelial progenitor cells, hypertension, senescence, autophagy, rhynchophylline

## Abstract

The increase of blood pressure accelerates endothelial progenitor cells (EPCs) senescence, hence a significant reduction in the number of EPCs is common in patients with hypertension. Autophagy is a defense and stress regulation mechanism to assist cell homeostasis and organelle renewal. A growing number of studies have found that autophagy has a positive role in repairing vascular injury, but the potential mechanism between autophagy and senescence of EPCs induced by hypertension has rarely been studied. Therefore, in this study, we aim to explore the relationship between senescence and autophagy, and investigate the protective effect of rhynchophylline (Rhy) on EPCs. In angiotensin II (Ang II)-treated EPCs, enhancing autophagy through rapamycin mitigated Ang II-induced cell senescence, on the contrary, 3-methyladenine aggravated the senescence by weakening autophagy. Similarly, Rhy attenuated senescence and improved cellular function by rescuing the impaired autophagy in Ang II-treated EPCs. Furthermore, we found that Rhy promoted autophagy by activating AMP-activated protein kinase (AMPK) signaling pathway. Our results show that enhanced autophagy attenuates EPCs senescence and Rhy rescues autophagy impairment to protect EPCs against Ang II injury.

## Introduction

Hypertension is a cardiovascular syndrome which can cause structure and function changes of the heart and blood vessel. Vascular injury and dysfunction are both the results of target organ injury and the etiology of hypertension (Bleakley et al., 2015). Vascular endothelium mainly regulates vasomotor function and vascular remodeling, and endothelial dysfunction is one of the most important reasons for the hypertension and target organ damage. While the elevated blood pressure (BP), in turn, further aggravates damage of endothelial cells and blood vessel. Endothelial progenitor cells (EPCs) are the precursor cells of endothelial cells, which mainly exist in bone marrow, cord blood, and peripheral blood (Murohara et al., 2000). EPCs are quite capable of repairing damaged vascular endothelium and regenerating blood vessel (Altabas et al., 2016; Kiewisz et al., 2016). Therefore, EPCs have a broad prospect in the research and treatment of cardiovascular and cerebrovascular diseases, diabetes, peripheral vascular diseases, and other vascular injury diseases.

EPCs constitute an important back-up system to maintain the integrity of endothelial cells and prevent the activation of endothelial cells (van Zonneveld and Rabelink, 2006). When the number of EPCs is reduced or the function is impaired, the abilities of EPCs to differentiate into mature endothelial cells and promote angiogenesis are weakened, and the endogenous repair function cannot be normally played, which aggravating vascular endothelial injury. While all aspects of the pathological mechanism of hypertension, including renin-angiotensin-aldosterone system (RAAS), autonomic nervous system, inflammation, and immune system abnormalities, can result in impaired EPCs, of which angiotensin II (Ang II) is the main factor (Luo et al., 2016).

The vascular repair ability of EPCs is closely related with its quantity, however, some studies have confirmed that the number of EPCs in the peripheral blood of hypertension patients is dramatically reduced (Oliveras et al., 2008; Umemura et al., 2008; Rocha et al., 2018), and the proportion of senescence EPCs is increased (Imanishi et al., 2005; Giannotti et al., 2010). Cell senescence is an extremely complex pathophysiological process involving multiple genes under the regulation of cell cycle, that is, cells change from normal growth state to irreversible growth stagnation state. EPCs senescence leads to a substantial reduction in the EPCs (Assmus et al., 2003), therefore, how to attenuate EPCs senescence and antagonize damage of EPCs is important on vascular repair.

Autophagy is a process in which the soluble macromolecules or dysfunctional organelles of the body engulf itself into vesicles and fuse with lysosomes to form autolysosomes to degrade the contents. Autophagy, as a highly conserved protective procedure in the case of changes in the external environment of cells, has been widely involved in the pathological process of various diseases. Previous study has found that activating autophagy can reduce oxidative stress and improve endothelial vasodilation in spontaneous hypertensive rats (Li et al., 2018), suggesting that autophagy has a protective effect on endothelial cells. Further research is needed on the effects of autophagy regulation on EPCs and whether it can antagonize EPCs injury in hypertension and sequentially repair damaged blood vessels.

The rhynchophylla total alkaloid (RTA) is the major active ingredient of rubiaceae plant *Uncaria*, and rhynchophylline (Rhy) is the most abundant and main component of RTA. We have previously found that RTA has the effect of lowering BP and protecting endothelial cells, and the preliminary mechanism may attribute to decrease inflammatory reaction and regulate antithrombotic actions related genes (Li et al., 2015a; Li et al., 2015b). Moreover, RTA inhibits the prothrombotic state induced by inflammatory damage of endothelial cells, which associated with the activation of the thrombin-receptor signaling pathway to inhibit fibrin generation and deposition (Li et al., 2017). The present study aimed to investigate the protective effect and underlying mechanism of Rhy for senescence EPCs.

## Materials and Methods

### The Preparation of Rhynchophylline

Rhy (Shanghai standard biotechnology, China) was dissolved in dimethyl sulfoxide and then diluted with DMEM·F12. The solution was stored at 4 °C.

### Isolation of Mononuclear Cells and Cell Culture

The Ethics Committee of Shandong University of Traditional Chinese Medicine approved the experimental protocol used in this study. All methods were performed in accordance with the Guide for the Care and Use of Laboratory Animals (published by the US National Institutes of Health). The healthy male Wistar rats (4–6 weeks old) were provided by experimental animal center of Shandong University. According to the previous description (Asahara et al., 1997; Quaranta et al., 2014; Luo et al., 2016), mononuclear cells were isolated by density gradient centrifugation using lymphocyte separation medium (Tianjin haoyang biological products technology, China). Briefly, according to the manufacturer’s instructions, homogenate rinse solution was used to flush the bone marrow cavity of tibia and femur in rats, centrifuged for 10 min and the supernatant was removed. The cells were resuspended into single cell suspension by sample diluent and then were added to the separation solution. After centrifugation, the cells of endothelial progenitor cell layer were gathered and washed for 3 times. The cells were plated at 1 × 10^6^/cm^2^ density on a fibronectin-coated culture flask and were cultured in EGM-2MV (Lonza Group, CH), which includes endothelial basal medium-2 (EBM-2) and EGM-2 Single Quots containing 5% fetal calf serum, 0.4% recombinant human fibroblast growth factor-B, 0.1% vascular endothelial growth factor, 0.1% recombinant human epidermal growth factor, 0.1% insulin-like growth factor-1, 0.1% ascorbic acid, 0.1% GA1000, and 0.04% hydrocortisone. The culture medium was changed to remove the nonadherent cells after 72 h and then was changed every 48 h. Cell states were observed and photographed under inverted phase contrast microscope every day.

EPCs were incubated with 2 μmol·L^-1^ Ang II (Sigma-Aldrich, USA) as a control. Except for the normal control group (cultured only with EGM-2MV), other groups were cultured with drug for 1 h and then added Ang II. On the basis of our previous study, all cells were incubated for 24 h before analysis.

### Immunofluorescent Double Staining

The original generation of EPCs were inoculated on a 12-well plate. Cells were incubated with 40 μg·mL^-1^ 1,1’-dioctadecyl-3,3,3’,3’-tetramethylindocarbocyanine- labeled acetylated low-density lipoprotein (Dil-Ac-LDL, Guangzhou Yiyuan Biotech, China) for 4 h in dark. After fixed with 3% paraformaldehyde for 30 min, the cells were incubated with 10 μg·ml^-1^ fluorescein isothiocyanate-labeled Ulex europaeus agglutinin 1 (FITC-UEA-1, Sigma-Aldrich, USA) in dark for 1 h. The staining results were observed with fluorescence microscope.

### Senescence-Associated β-Galactosidase (SA-β-Gal) Activity Assay

EPCs were harvested and seeded in a 6-well plate. After adhered to the wall and fused about 80%, the cells were treated in different ways for 24 h. According to the manufacturer’s protocol of a Senescent Cells Staining Kit (Beyotime Institute of Biotechnology, China), the cells were fixed for 15 min (room temperature) in SA-β-gal stationary liquid, washed and incubated for 12 h at 37 °C (no CO_2_) with SA-β-gal stain solution. Total cells and stained cells were counted respectively.

### Telomerase Assay

Telomerase activity was measured by a telomeric repeat amplification protocol (TRAP) assay with the TeloTAGGG Telomerase PCR ELISA ^plus^ Kit (Roche Molecular Biochemicals, Germany). According to the manufacturer’s protocol, approximately 1 × 10^6^ cells were harvested for each reaction and centrifuged at 12,000 ×*g* for 20 min at 4 °C. TRAP reaction products were generated in the supernatant after amplification and incubated with the sealant at 37 °C for 1 h. Anti-DNP antibody was added and incubated for 30 min in dark. After adding TMB solution and termination solution, the absorbance for the final product was acquired by measuring absorbance at 450 and 690 nm. The difference value (OD = A450-A690) stands for telomerase activity.

### Western Blot Assay

After RIPA and PMSF were mixed in a ratio of 100:1, the cell lysis buffer was added into EPCs, and the supernatant was collected after centrifugation. Protein concentration was determined by BCA protein assay kit (Tiangen Biotech, China). Protein samples were separated by sodium dodecyl sulfate-polyacrylamide gel electrophoresis (SDS-PAGE) and transferred to polyvinylidene difluoride (PVDF) membranes (Millipore Corporation, USA). The membranes were sealed in 5% skim milk for 1 h and then incubated with following primary antibodies overnight at 4 °C: anti-p53, anti-LC3A/B, anti-Beclin-1, anti-phosphor-AMPKα, anti-AMPKα (1:1,000, Cell Signaling Technology, USA), anti-p21 (1:1,000, Santa Cruz Biotechnology, USA), anti-p62 (1:1,000, Abcam, USA), anti-β-actin (1:10,000, Proteintech Group, USA). On the second day, the membranes were incubated with horseradish peroxidase-conjugated secondary antibody (1:10,000) for 1 h. Bands were detected with Immobilon Western HRP Substrate (Millipore Corporation, USA), and the average densitometric was analyzed using ImageJ 1.8.0 software.

### mRFP-GFP-LC3 Immunofluorescence

The EPCs were inoculated at the concentration of 1 × 10^5^/ml into a 24-well plate. When the cell confluence rate was between 50–70%, the cells were transfected the autophagy double-labeled adenovirus mRFP-GFP-LC3 (Hanbio Biotechnology, China) in 37 °C for 2 h and then were cultured with EGM-2MV for 12 h. After exposure to drugs and Ang II for another 24 h, the cells were observed by fluorescence microscope. Furthermore, the number of red dots, green dots, yellow dots, and free red dots in at least three different images were counted.

### Transmission Electron Microscopy

The EPCs were fixed in 3% glutaraldehyde solution and 1% osmium acid solution for 2 and 1 h respectively, dehydrated by acetone solution, and embedded in Epon812. The embedded blocks were sliced into semi-thin sections, dyed with toluidine blue, incised into ultra-thin sections under the light microscope by an LKB-V ultra-thin slicing machine, stained with uranium acetate and lead citrate, and then were observed by the JEOL-1200EX transmission electron microscope.

### Proliferative Activity Assay

According to the instruction of EdU incorporation assay kit (Guangzhou Ribobio, China), EPCs in logarithmic growth phase were inoculated into a 96-well plate and cultured to the normal growth stage. The EdU solution was diluted by EGM-2MV to the EdU medium of 50 μM. Add EdU medium to each well and incubate at 37 °C for 4 h. Add 4% paraformaldehyde and incubate at room temperature for 30 min. Add 2 mg·ml^-1^ glycine and wash in decolorizing shaker for 5 min. Add 1× Apollo^®^ staining reaction solution to each well and incubate in dark for 30 min with oscillation. Add 1× Hoechst33342 and incubate in dark for 30 min with oscillation. The staining results were observed with fluorescence microscope.

### Migration Assay

Cell migration rate was determined using a transwell chamber (Coning, USA). EPCs were diluted to 1 × 10^5^/ml with EBM-2. 0.5 ml EGM-2MV was added to lower compartment, and 0.2 ml cell suspension was added to upper compartment and incubated for 6 h at 37 °C. The culture medium was sucked out, and the remaining cells on the upper side of the filter membrane were gently wiped with a dry cotton swab. The cells were fixed with 4% paraformaldehyde for 30 min and stained with hematoxylin staining solution and eosin staining solution for 30 and 10 min respectively. The number of EPCs migrated from the upper compartment to the lower compartment was counted.

### Adhesion Assay

Fibronectin was diluted with DMEM·F12 (the ratio is 1:9), and tiled in a 24-well plate for 12 h at 4°C. After different treatments, EPCs were harvested, inoculated on the 24-well plate, and cultivated for 1 h at cell incubator. Phosphate-buffered saline was used to remove the unattached EPCs. The cells were fixed with 4% paraformaldehyde for 10 min, stained with hematoxylin staining solution and eosin staining solution for 30 and 10 min respectively. The number of adherent cells was counted under the microscope.

### *In Vitro* Network Formation Assay

Network formation capacity was detected using a tube formation assay kit (Sigma-Aldrich, USA). 0.05 ml BME/per well was added into a 96-well plate, and cultivated for 1 h in cell incubator. EPCs were diluted to 1 × 10^5^/ml with EBM-2 and 0.1 ml cell suspension was added to 96-well plate. After 6 h, number of tubular structures was counted.

### Transfection of Small-Interfering RNA (siRNA) and Quantitative Real-Time PCR (qRT-PCR)

The EPCs were transfected with 100 nM control siRNA (sc-36869, Santa Cruz Biotechnology, USA) or 100 nM AMPKα siRNA, containing 50 nM AMPKα1 siRNA (sc-270142, Santa Cruz Biotechnology, USA) and 50 nM AMPKα2 siRNA (sc-155985, Santa Cruz Biotechnology, USA). According to the manufacturer’s specification, when the cell confluence rate was between 60–80%, siRNA duplex and siRNA transfection reagent were diluted with siRNA transfection medium. siRNA duplex solution and dilute transfection reagent were mingled and then incubated 30 min at room temperature. The cells were washed with siRNA transfection medium, overlaid by the siRNA transfection reagent mixture, and incubated 6 h in incubator. Removed the transfection mixture, replaced with normal growth medium and incubated for an additional 48 h. The expression level of AMPKα was checked to determine if the knockdown was successful.

AMPKα mRNA expression was detected by qRT-PCR. Total RNA of EPCs was extracted by TRIzol method and reverse-transcribed into cDNA using the PrimeScript RT reagent kit (Takara Biomedical Technology, China). The specific forward/reverse primers (Sangon Biotech, China) matching the published sequences were as follows: AMPKα (5’-CCTTTACCACGGTTGATTTCTC-3’; 5’-CAGGCTCTACTTTGATCGCACT-3’) and β-actin (5’-CGTTGACATCCGTAAAGA-3’; 5’-AGCCACCAATCCACACAG-3’). The qRT-PCR was performed with LightCycler^®^480 SYBR Green I Master kit. The reaction mixture contained LightCycler^®^480 SYBR Green I Master, forward/reverse primers, cDNA and DNase/RNase-free ddH_2_O. The samples were detected by Roche LightCycle^®^480 and each sample was tested in triplicate.

### Statistical Analysis

SPSS statistics 22.0 software was used for statistical analysis of the data from at least 3 independent experiments. All results were expressed as mean ± standard deviation (S.D.). The two-tailed paired Student’s *t*-test was used for comparison between two groups. Differences were considered statistically significant at *P* < 0.05.

## Results

### Characterization of Rat Bone Marrow-Derived EPCs

**Figure 1:**
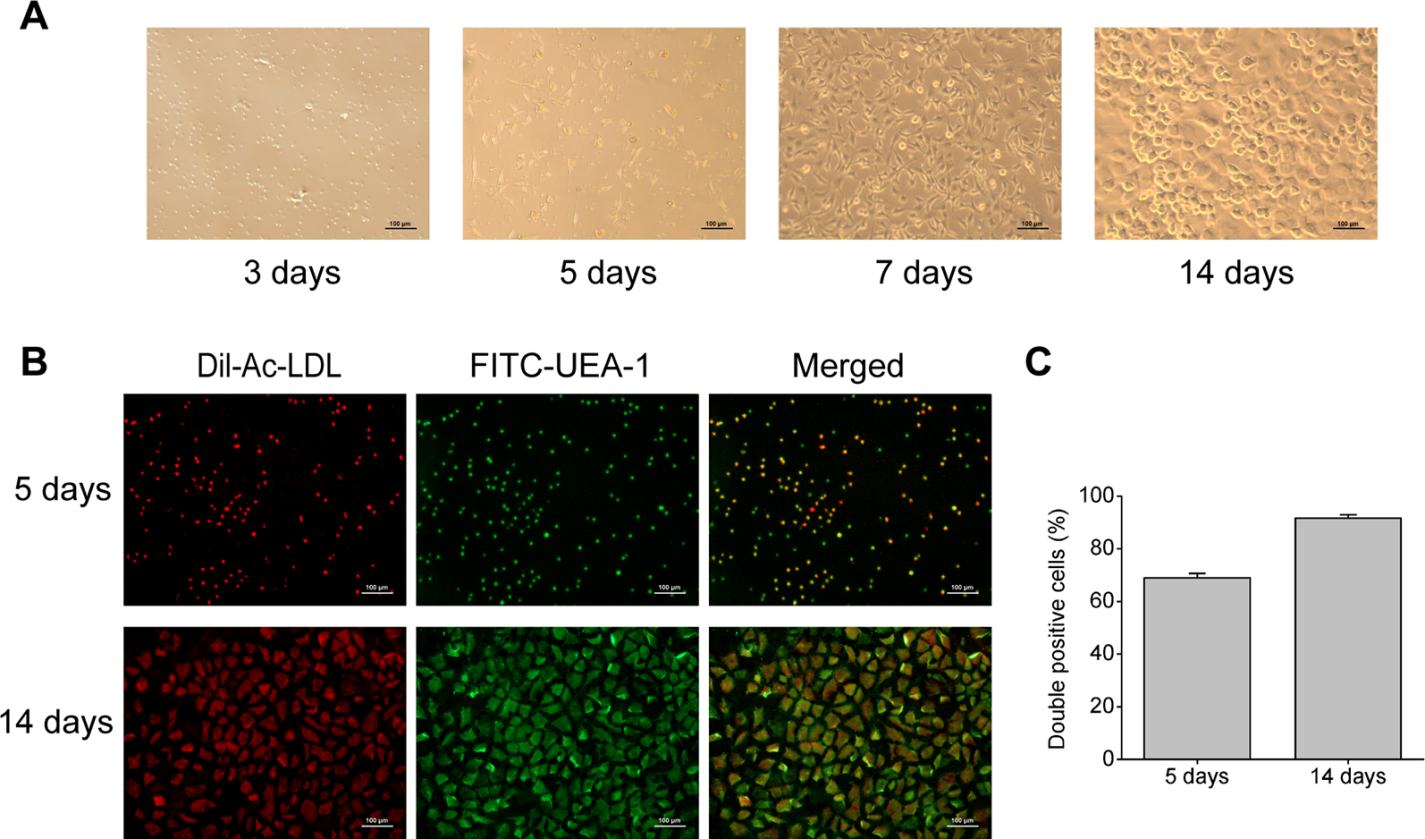
Characterization of rat bone marrow-derived EPCs. **(A)** Representative images of rat bone marrow-derived EPCs on the 3rd, 5th, 7th, and 14th day of culture (100× magnification). **(B)** Immunofluorescent images indicating Dil-Ac-LDL (red) incorporation and FITC-UEA-1 (green) binding by EPCs (100× magnification). **(C)** Quantitative analysis of the double positive cells.

Rat bone marrow- derived EPCs have typical morphology of spindle-shaped and paving stone form, and can form tubular structure (**Figure 1A**), which is consistent with EPCs reported in literature (Sekiguchi et al., 2011). Immunofluorescent double staining is a functional identification method of EPCs, and also one of the most commonly used identification methods. After staining with Dil-Ac-LDL and FITC-UEA-1, the double positive cells were differentiated EPCs, showing orange or yellow (**Figure 1B**). And as shown in **Figure 1C**, cultured for 5 days, the ratio of double positive cells reached 69%, and the ratio rose to 92% at 14 days. This result means EPCs induced by culture can endocytose Ac-LDL and combine with UEA-1, indicating that EPCs have the ability to differentiate into endothelial cells.

### Ang II Triggered Off EPCs Senescence and Inhibited EPCs Autophagy

**Figure 2:**
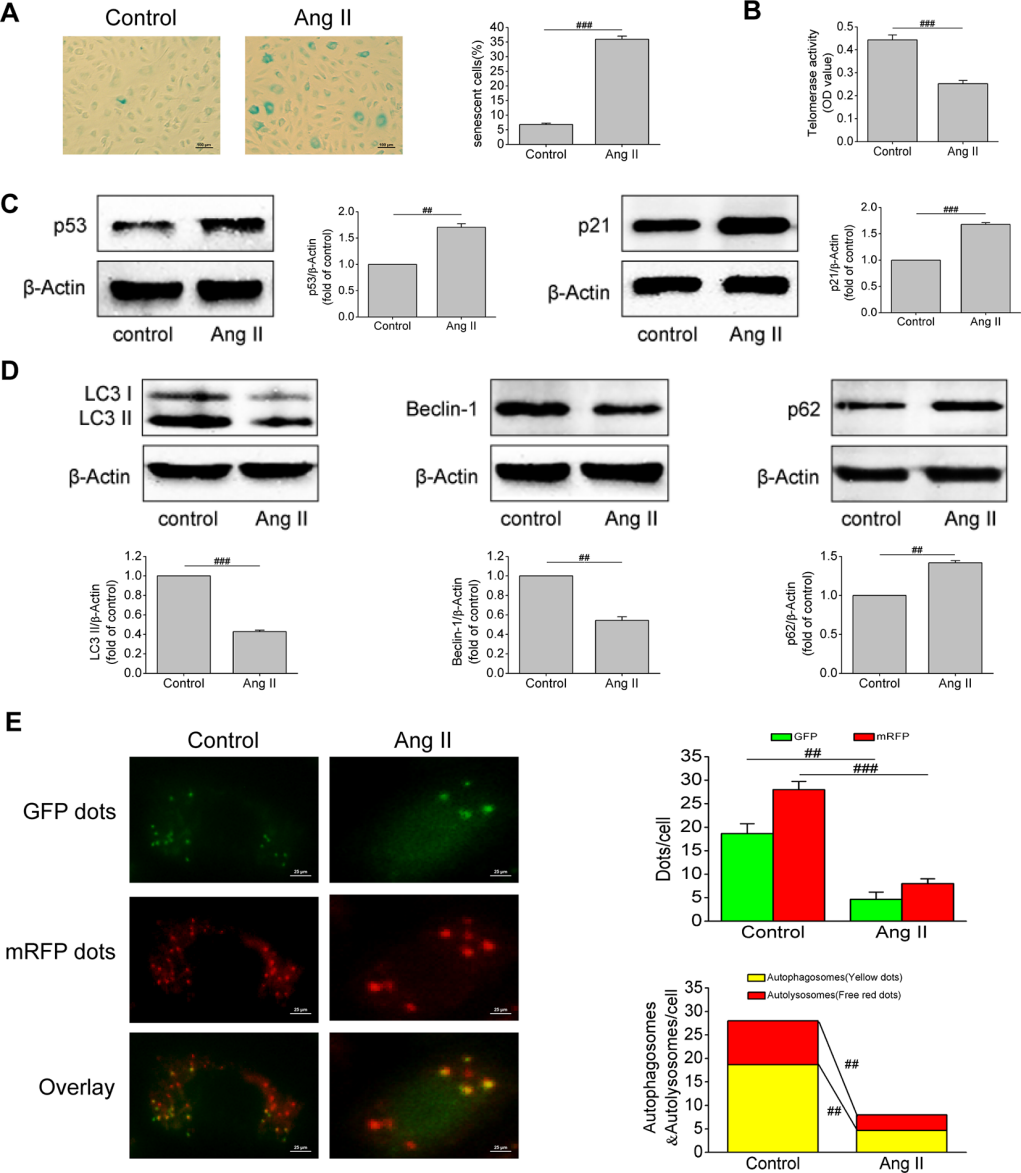
Ang II triggered off EPCs senescence and inhibited EPCs autophagy. **(A)** Representative images and quantitative analysis of SA-β-gal activity assay (100× magnification). **(B)** Telomerase activity was determined by telomeric repeat amplification protocol assay and indicated by the difference value (OD = A450-A690). **(C)** Western blot analysis of the expression of p53 and p21 in EPCs in the absence or presence of Ang II. The data was presented in fold of control. **(D)** Western blot analysis of the expression of LC3-II, beclin-1 and p62 in EPCs in the absence or presence of Ang II. The data was presented in fold of control. **(E)** Representative images and quantitative analysis of mRFP-GFP-LC3 immunofluorescence (400× magnification). All data were pooled as mean ± S.D. (error bars) from three independent biological repeats experiments, each of which included 4 technical repeats. ^##^P < 0.01; ^###^P < 0.001.

Ang II is an important component of the RAAS and one of the strongest vasoconstrictor active substances currently known. It has been found that Ang II can cause vascular injury, but its effect on EPCs has been rarely studied. Based on this, we first examined the effect of Ang II on EPCs senescence. Results of SA-β-gal activity assay revealed that the volume of senescent EPCs increased, the edge of cells was blurred, and the cytoplasm was stained. After Ang II intervention, the proportion of senescent EPCs increased (*P* < 0.001, **Figure 2A**). Severely shortened telomere is a signal of cell aging. Ang II inhibited telomerase activity (*P* < 0.001, **Figure 2B**), indicating that Ang II shortened telomere length. Furthermore, Ang II increased the levels of p53 and p21, which are both important molecular markers of cell senescence (*P* = 0.003 and *P* < 0.001, respectively; **Figure 2C**). Afterwards, we examined changes in autophagy of EPCs after Ang II intervention. Ang II decreased the levels of LC3-II and beclin-1 but increased the level of p62, meaning Ang II inhibited autophagy (*P* < 0.001, *P* = 0.002, and 0.0015, respectively; **Figure 2D**). Furthermore, the number of autophagosomes and autolysosomes in cells was detected by mRFP-GFP-LC3 immunofluorescence to determine the formation of autophagosomes and the level of autophagic flux. After Ang II exposure, the numbers of green dots, red dots, yellow dots (autophagosomes), free red dots (autolysosomes) were all reduced (*P* = 0.002, 0.0008, 0.002, 0.009, respectively; **Figure 2E**). The results demonstrated Ang II inhibited the formation of autophagosomes and autophagic flux. These results suggested that Ang II induced EPCs senescence and inhibited EPCs autophagy.

### Enhancing Autophagy Attenuated Ang II-Induced EPCs Senescence

**Figure 3:**
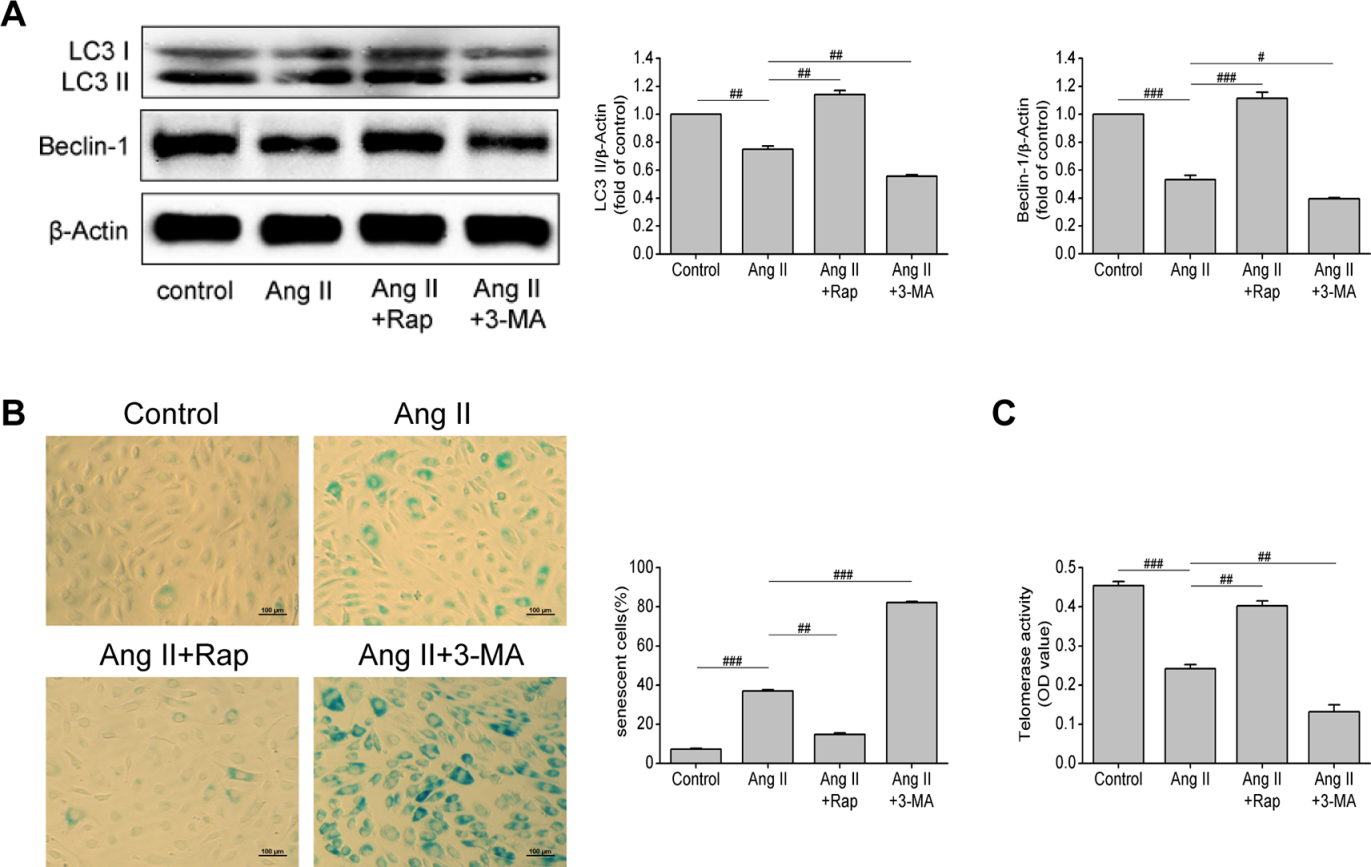
Enhancing autophagy attenuated Ang II-induced EPCs senescence. **(A)** Western blot analysis of LC3-II and beclin-1 in EPCs in indicated groups. The data was presented in fold of control. **(B)** Representative images and quantitative analysis of SA-β-gal activity assay (100× magnification). **(C)** Telomerase activity was determined by telomeric repeat amplification protocol assay and indicated by the difference value (OD = A450-A690). All data were pooled as mean ± S.D. (error bars) from three independent biological repeats experiments, each of which included 3 technical repeats. ^#^P < 0.05; ^##^P < 0.01; ^###^P < 0.001.

Through the previous experiments, we found that Ang II can simultaneously affect senescence and autophagy of EPCs, so we questioned whether there is a link between senescence and autophagy of EPCs. To further verify the relationship between autophagy and cell senescence, we used rapamycin (Rap, an inhibitor of mTOR) and 3-methyladenine (3-MA, an inhibitor of autophagy) to motivate or inhibit autophagy, so as to observe the changes in the senescence degree of EPCs. As shown in **Figure 3A**, the levels of LC3-II and beclin-1 were increased by Rap (*P* = 0.005 and *P* < 0.001, respectively), but decreased by 3-MA (*P* = 0.005 and 0.013, respectively), reflecting that Rap rescued the impaired autophagy in Ang II-treated EPCs, while 3-MA aggravated the damage and led to the lower levels of autophagy. The intervention of Rap brought down the proportion of senescent EPCs (*P* = 0.001, **Figure 3B**) and elevated telomerase activity (*P* = 0.006, **Figure 3C**), while the 3-MA had the opposite effect. Rap restrained Ang II-induced EPCs senescence and 3-MA further expedited senescence on the basis of Ang II. Based on the above experiments, we draw a conclusion that Rap attenuated Ang II-induced EPCs senescence by enhancing autophagy but 3-MA exerted the opposite effect, which accelerated senescence by inhibiting autophagy.

### Rhynchophylline Enhanced Autophagy and Attenuated Ang II-Induced EPCs Senescence

**Figure 4:**
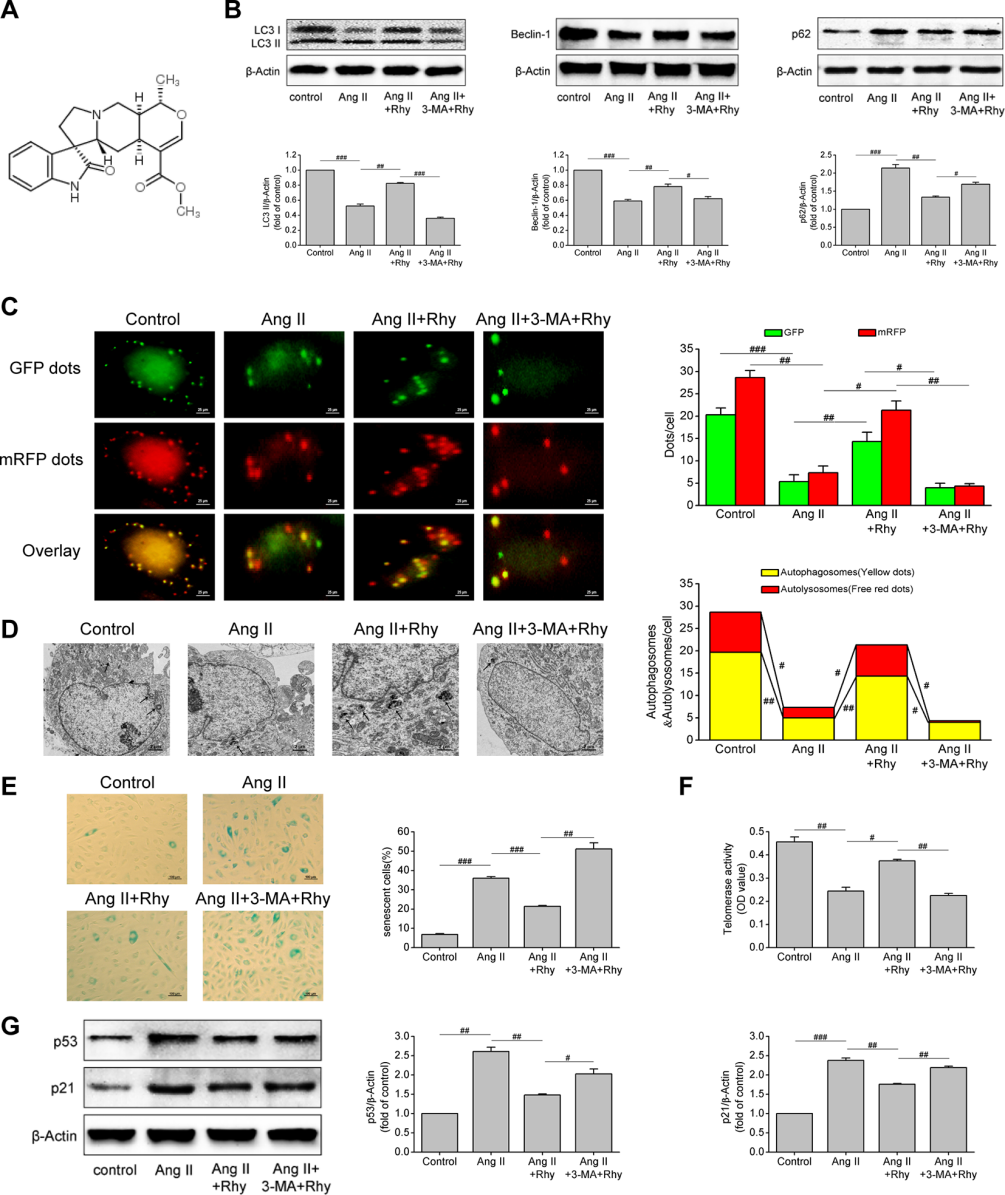
Rhynchophylline enhanced autophagy and attenuated Ang II-induced EPCs senescence. **(A)** The chemical structure of Rhy. **(B)** Western blot analysis of LC3-II, beclin-1 and p62 expression in EPCs in indicated groups. The data was presented in fold of control. **(C)** Representative images and quantitative analysis of mRFP-GFP-LC3 immunofluorescence (400× magnification). **(D)** Autophagosomes were observed by transmission electron microscopy. **(E)** Representative images and quantitative analysis of SA-β-gal activity assay (100× magnification). **(F)** Telomerase activity was determined by telomeric repeat amplification protocol assay and indicated by the difference value (OD = A450-A690). **(G)** Western blot analysis of p53 and p21 expression in EPCs in indicated groups. The data was presented in fold of control. All data were pooled as mean ± S.D. (error bars) from three independent biological repeats experiments, each of which included 4 technical repeats. ^#^P < 0.05; ^##^P < 0.01; ^###^P < 0.001.

The molecular formula of Rhy is C_22_H_28_N_2_O_4_, and its chemical structure is shown in **Figure 4A**. Our previous studies have confirmed the protective effect of Rhy on vascular endothelial cells in spontaneous hypertensive rats. Therefore, to further observe the effects of Rhy on EPCs autophagy and senescence, we added Rhy intervention in Ang II-treated EPCs. Rhy rescued autophagy impaired by Ang II, as indicated by the increased LC3-II content (*P* = 0.005), beclin-1 content (*P* = 0.002), and also the decreased p62 level (*P* = 0.009), but 3-MA reversed the effects of Rhy (**Figure 4B**). According to the results of mRFP-GFP-LC3 immunofluorescence, after Rhy intervention, the red dots, green dots, yellow dots, and free red dots which were decreased by Ang II were increased, meaning Rhy promoted the formation of autophagosomes and the level of autophagic flux. While the promotion to autophagy by Rhy was dramatically counteracted by 3-MA (**Figure 4C**). The same conclusion was obtained by observing autophagosomes with transmission electron microscope. After Ang II exposure, fewer autophagosomes and more lipid droplets were found. Rhy restrained this phenomenon, but 3-MA played an opposite role (**Figure 4D**). Through further detection of cell senescence related indicators, it was found that Rhy reduced the proportion of senescent cells (*P* < 0.001, **Figure 4E**), increased the activity of telomerase (*P* = 0.011, **Figure 4F**), and reduced the levels of p53 and p21 (*P* = 0.003 and 0.001, respectively; **Figure 4G**). The anti-senescence effect of Rhy can be destroyed by autophagy inhibitor 3-MA, meaning that inhibition of autophagy prevented Rhy from attenuating cell senescence. These data suggested that Rhy attenuated Ang II-induced EPCs senescence by enhancing autophagy.

### Rhynchophylline Improved EPCs Function

**Figure 5:**
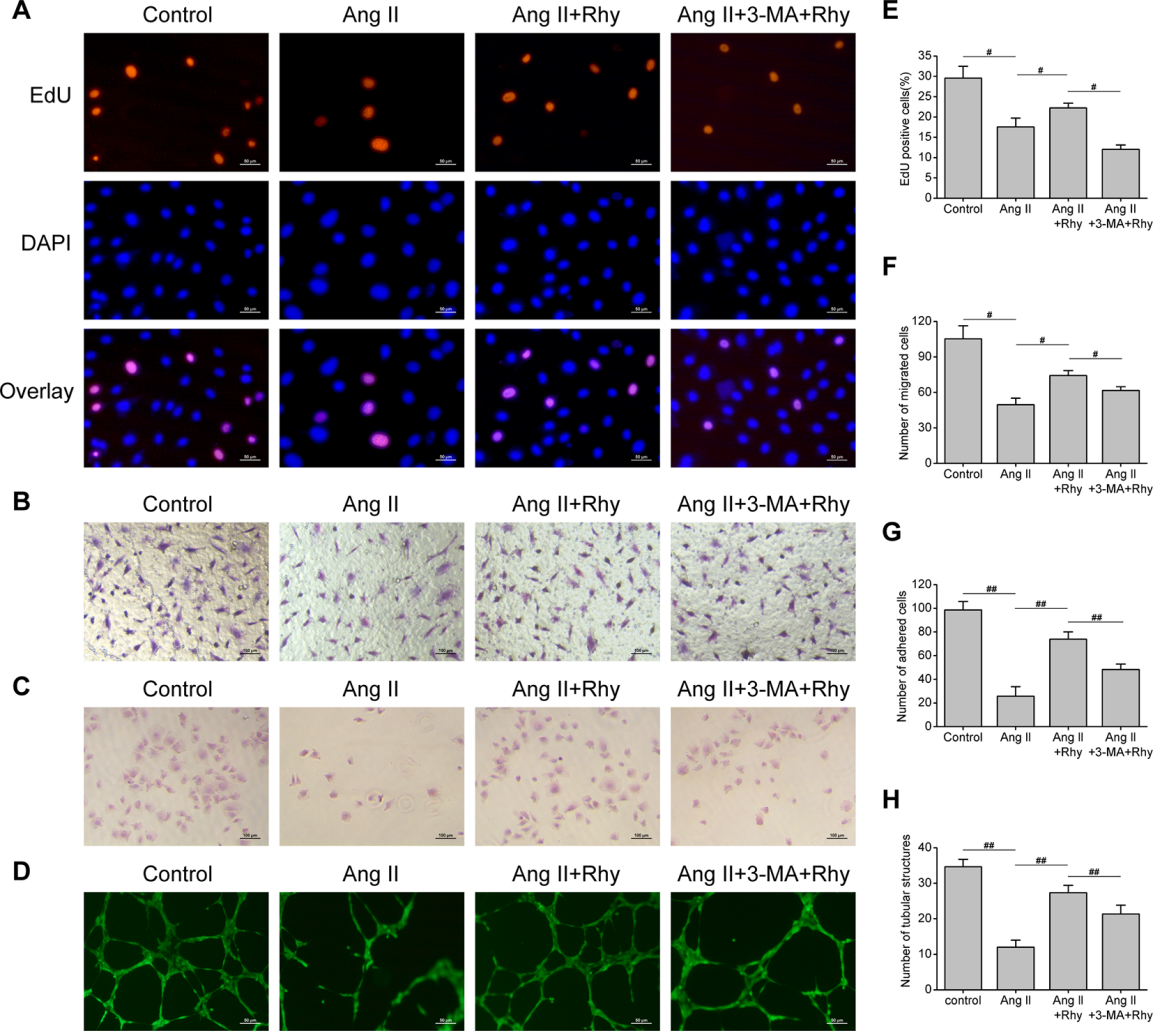
Rhynchophylline improved EPCs function. **(A, E)** Representative images and quantitative analysis of proliferative activity assay (200× magnification). **(B, F)** Representative images and quantitative analysis of migration assay (H&E stain, 100× magnification). **(C, G)** Representative images and quantitative analysis of adhesion assay (H&E stain, 100× magnification). **(D, H)** Representative images and quantitative analysis of network formation assay *in vitro* (200× magnification). The number of tubular structures in the field represented the angiogenesis capacity of EPCs. All data were pooled as mean ± S.D. (error bars) from three independent biological repeats experiments, each of which included 3 technical repeats. ^#^P < 0.05; ^##^P < 0.01.

The function of EPCs to repair blood vessels is closely related to its quantity, migration, adhesion and angiogenesis, thus we examined the effect of Rhy on these functions. The results showed that Ang II inhibited DNA replication activity (**Figures 5A, E**), and the numbers of migrated cells (**Figures 5B, F**), adhered cells (**Figures 5C, G**), and tubular structures (**Figures 5D, H**) were reduced. Rhy increased DNA replication activity, improved migratory and adherent capacities, and promoted the formation of tubular structures. Whereas the improvement effect was not obvious in the group treated with Rhy and 3-MA. These results suggested that Rhy improved the cellular function damaged by Ang II, which associated with its effect of increased autophagy.

### Rhynchophylline Enhanced Autophagy by Activating AMPK Signaling Pathway

**Figure 6:**
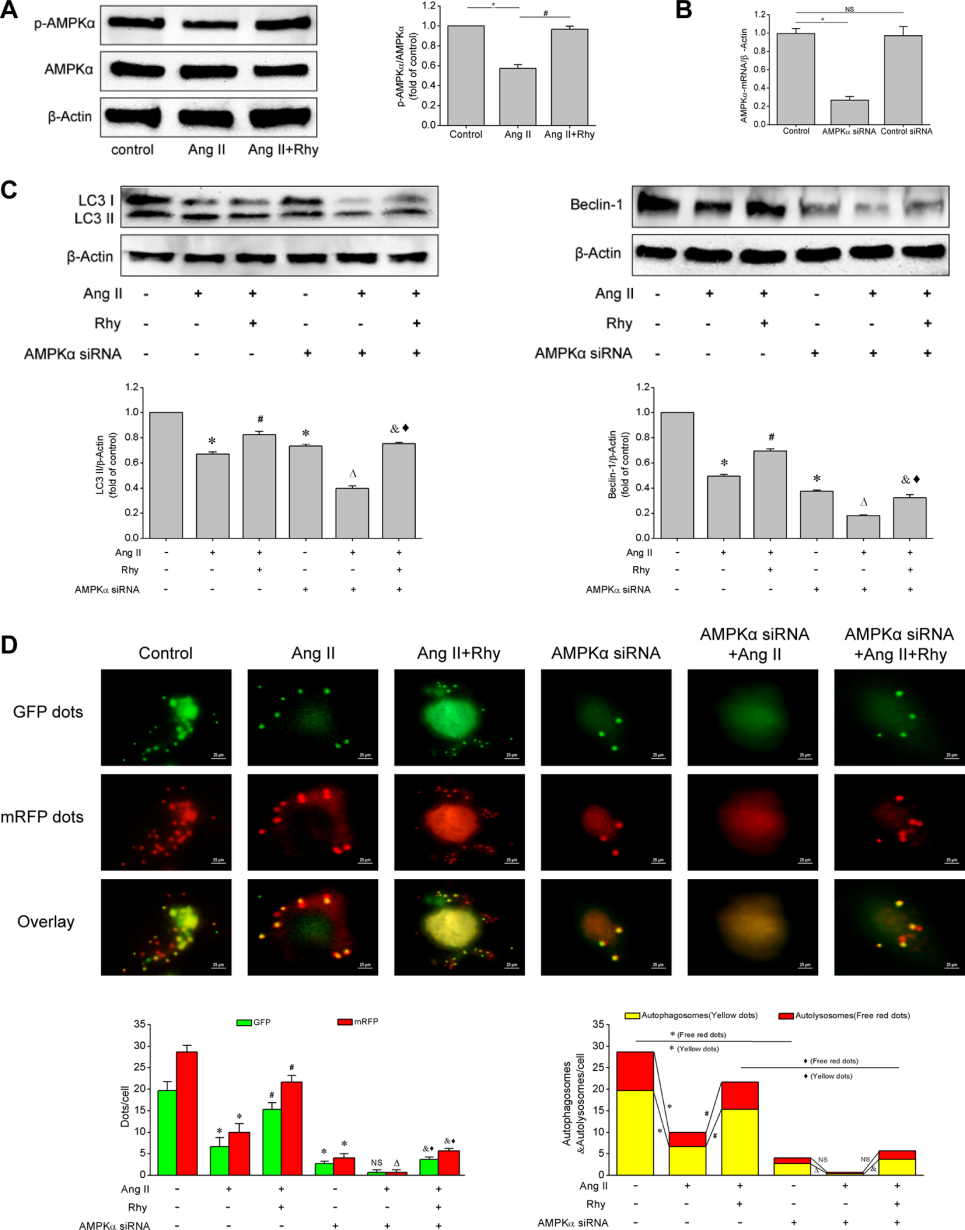
Rhynchophylline enhanced autophagy by activating AMPK signaling pathway. **(A)** Western blot analysis of p-AMPKα and AMPKα expression in EPCs in indicated groups. The data was presented in fold of control. **(B)** The mRNA expression of AMPKα detected by qRT-PCR in indicated groups. **(C)** Western blot analysis of LC3-II and beclin-1 expression in EPCs in indicated groups. The data was presented in fold of control. **(D)** Representative images and quantitative analysis of mRFP-GFP-LC3 immunofluorescence (400× magnification). All data were pooled as mean ± S.D. (error bars) from three independent biological repeats experiments, each of which included 3 technical repeats. ^*^P < 0.05 vs control group; ^#^P < 0.05 vs Ang II group; ^Δ^P < 0.05 vs AMPKα siRNA group; ^&^P < 0.05 vs AMPKα siRNA+Ang II group; ^⋄^P < 0.05 vs Ang II+Rhy group.

Based on the above experiments, we known that Rhy attenuated EPCs senescence and improved cellular function, which was related to the enhancement of autophagy. To further clarify how Rhy regulates autophagy, we examined the activation status of AMPK signaling pathway. Rhy increased the level of p-AMPKα in Ang II-treated EPCs (*P* < 0.001, **Figure 6A**), which reflected that Rhy promoted the occurrence of autophagy by regulating AMPK signaling pathway. To attain more convincing results, we used AMPKα siRNA to knock down AMPKα. The result of qRT-PCR showed that the AMPKα had been knocked out after the transfection with siRNA (**Figure 6B**). As shown in **Figures 6C, D**, after AMPK pathway was partly deactivated, the LC3-II and beclin-1 expression were decreased, and the formation of autophagosomes was inhibited. By comparing Ang II+Rhy group with AMPKα siRNA+Ang II+Rhy group, we can infer that the blocking of AMPK pathway greatly affected the positive role of Rhy in promoting autophagy, in other words, the AMPK pathway was relevant to the promotion of autophagy by Rhy. The results suggested that Rhy enhanced autophagy by activating AMPK signaling pathway.

## Discussion

EPCs senescence and hypertension are a reciprocal causation. Autophagy has a protective effect on vascular endothelium and its effect on regulating aging has been proved in recent years, but the impact of autophagy in EPCs senescence caused by hypertension remains unclear. This study demonstrated a potential link between senescence and autophagy of EPCs, and revealed the protective effect of Rhy on EPCs. Enhanced autophagy by Rap can inhibit Ang II-induced EPCs senescence, while inhibition of autophagy by 3-MA will further aggravate cell senescence. Rhy has a positive effect on promoting autophagy and attenuating EPCs senescence, which is similar to Rap. At the same time, we found that the effect of Rhy on attenuating senescence and improving cell function was observably inhibited by 3-MA, which reflected that its effect was related to the promotion of autophagy. In addition, Rhy enhanced autophagy by activating AMPK signaling pathway.

Endothelial dysfunction plays an important role in the occurrence and development of hypertension. EPCs are a kind of pluripotent stem cells and a kind of precursor cells that have not yet differentiated into mature vascular endothelial cells (Asahara et al., 1997). In recent years, it has been found that circulating EPCs can independently predict the progression of atherosclerosis and other cardiovascular events, such as acute myocardial infarction and atherothrombotic stroke, thus are considered as a marker of cardiovascular disease (Cuadrado-Godia et al., 2015; Shimoni et al., 2016). EPCs have a strong ability to proliferate and differentiate, and are involved in the repair of vascular endothelium (Wang et al., 2013). EPCs also paracrine various factors that promote vascular repair (Mudyanadzo, 2018), which play a role in maintaining the integrity of vascular endothelial structure and function. In addition, EPCs motivate vascular regeneration and can be used as a potential therapeutic tool for cardiovascular ischemic diseases (Bueno-Beti et al., 2019).

EPCs play an active role in improving tissue ischemic and motivating vascular repair, but obesity and cardiovascular diseases threaten the function, senescence, and trans-differentiation of EPCs (Goligorsky, 2014; Mudyanadzo, 2018). Furthermore, various clinical conditions including hypertension underlie EPCs dysfunction, and hypertension can be used as the most important independent predictor of EPCs dysfunction (Vasa et al., 2001). Increased studies have shown a decrease in EPCs content in peripheral blood of patients with hypertension, and EPCs function was inversely correlated with BP (Suzuki et al., 2014). The proliferation and migration activity of circulating EPCs in hypertensive patients were prominently lower than those with normal BP, and the impaired activity was associated with decreased arterial elasticity (Yang et al., 2010). Moreover, both hypertensive rats and hypertensive population showed decreased telomerase activity and increased number of aging EPCs (Imanishi et al., 2005), which brought about EPCs dysfunction (Zhao et al., 2010). In addition to the continuous increase of autonomic nervous system sympathetic excitation (Zubcevic et al., 2014) and inflammatory response (Eirin et al., 2013), the excessive activation of RAAS can cause the imbalance of reactive oxygen species (ROS) and nitric oxide (NO), resulting in the reduction of EPCs and dysfunction (Thum et al., 2011). Ang II, an important component of RAAS, can not only directly damage EPCs, but induce inflammatory response to aggravate EPCs dysfunction (Luo et al., 2016). Increased BP leads to decreased quantity and impaired function of EPCs, which makes endothelial injury unable to be well repaired and in turn further increases BP. Therefore, how to inhibit EPCs damage caused by hypertension is of great significance for BP control and target organ protection.

EPCs are not only derived from bone marrow, but also from spleen, vascular outer membrane, and even some endothelial cells themselves, moreover, they have different tendency to differentiate and repair tissues (Kamei et al., 2017; Rana et al., 2018). Even so, bone marrow-derived EPCs are still the main source of EPCs in the peripheral circulation. Accordingly, in this study, EPCs were extracted from bone marrow of Wistar rats for follow-up experiments. Cell senescence is considered to be one of the important reasons leading to depression and dysfunction of EPCs. Since Ang II is the main factor of EPCs injury in hypertension, we first examined the effect of Ang II on EPCs senescence. Senescent cells can specifically express β-galactosidase, which is the most widely used biomarker of senescent cells. Moreover, telomere length and stability are closely related to cell senescence. Telomerase can prolong the shortened telomere and keep telomere from being depleted by cell division, thus enhancing the proliferation ability of cells *in vitro*. P53 and p21 are important molecular markers of cell senescence, which can regulate cell cycle and senescence from multiple aspects. The upregulation of p21 expression notably inhibits the activity of cyclin dependent kinase, making a large number of cells stay in G1 phase of the cell cycle, thus inducing and accelerating cell senescence. Our results showed that Ang II induced EPCs senescence, as indicated by the increased β-galactosidase activity, the decreased telomerase activity, higher p53 and p21 expression.

Autophagy is not only the major intracellular degradation system, but a dynamic recycling system, which can degrade cellular components and provide energy for cell survival, so as to cope with environmental and cellular stress. In recent years, it has been found that autophagy plays an important role in cardiovascular diseases (Mei et al., 2015). Autophagy protects heart and preserves endothelial function by increasing NO bioavailability, and reducing oxidative stress and inflammation (LaRocca et al., 2012; Chen et al., 2013). We examined the effect of Ang II on autophagy to determine the autophagy status in hypertension. LC3 turnover is one of the principal markers for monitoring autophagy. In the process of cell autophagy, LC3 is cut by Atg4 to form LC3-I, then LC3-I and phosphatidyl ethanolamine covalently connect and insert into the membrane of autophagosome, that is LC3-II. Moreover, beclin-1 is also involved in the formation and extension of autophagosome. Accordingly, the elevated levels of LC3-II and beclin-1 indicate that the number of autophagosomes is increased. It is worth noting that the level of LC3-II should be compared with actin, but not with LC3-I (Klionsky et al., 2016). P62 is a key protein for the formation of autolysosome. When autophagic flux is expedite, that is, autolysosomes can form and then degrade the damaged organelles and longevity proteins, the content of p62 will decrease. The detection of mRFP-GFP-LC3 immunofluorescence reflects the process of autophagy, including the formation of autophagosomes and the level of autophagic flux. Autophagosomes are labeled by both mRFP and GFP, thus appear yellow. Nevertheless, when autophagosomes fuse with lysosomes to form autolysosomes, the acidic environment inside the lysosomes quenches the fluorescent signal of GFP, therefore, autolysosomes are only labeled with red. In our study, after the intervention by Ang II, the levels of LC3-II and beclin-1 were decreased, while p62 content was increased. Moreover, the number of autophagosomes and autolysosomes of Ang II-treated EPCs was markedly lower than the normal cells, indicating decreased autophagic flux. The results demonstrated Ang II down-regulated the level of autophagy in EPCs.

Autophagy plays a crucial role in prolonging longevity, and it has been proposed that autophagy is bound up with organ aging (Sasaki et al., 2017) and cell senescence (Levine and Kroemer, 2008). Damaged organelles and abnormal proteins accumulate continuously as cells age, while autophagy maintains tissue homeostasis by degrading these substances. Autophagy decreases with age, and the downregulation of autophagy promotes the aging of heart and blood vessel (Shirakabe et al., 2016). Autophagy defect caused by gene deletion can also cause shortened lifespan and accelerated organ aging, including heart, lung, and spleen (Fang et al., 2019). Moreover, lysosomal membrane permeabilization and subverted autophagy in embryonic EPC lead to stress-induced premature senescence (Chen et al., 2012). Autophagy has an inhibitory effect on aging, but the role of autophagy in hypertension is controversial. Therefore, we analyzed the effect of autophagy on Ang II-induced EPCs senescence. We found that enhancing autophagy through Rap mitigated Ang II-induced cell senescence, on the contrary, 3-MA aggravated senescence by weakening autophagy.

Our previous studies have demonstrated that RTA has the effect of lowering BP and protecting endothelial cells. This study further explored the effect of Rhy, the main component of RTA, on EPCs senescence and autophagy. Rhy up-regulated LC3-II and beclin-1 expression and down-regulated p62 expression, indicating Rhy rescued the impaired autophagy in Ang II-treated EPCs. The same conclusion can be reflected by the increased autophagosomes and autophagic flux in mRFP-GFP-LC3 immunofluorescence and transmission electron microscopy. In addition, after culturing with Rhy, the induction effect of Ang II on EPCs senescence was inhibited, which was manifested as decreased β-galactosidase activity, increased telomerase activity, and decreased p53 and p21 expression. Therefore, we draw a conclusion that Rhy has an active role in regulating senescence and autophagy of EPCs. To further verify whether Rhy inhibits EPCs senescence by enhancing autophagy, we used 3-MA to inhibit the promoting effect of Rhy on autophagy. The results showed that when the function of enhancing autophagy was suppressed, Rhy could not delay the senescence, but further aggravated the EPCs senescence caused by Ang II. In summary, our study suggested that autophagy defects aggravated cell senescence, and Rhy enhanced autophagy to attenuate EPCs senescence.

Normally, most EPCs are dormant and contact with stromal cells in bone marrow. When the endothelium is damaged, the connection between EPCs and stromal cells is weakened due to the influence of some inflammatory cytokines and mobilization factors, such as tumor necrosis factor-α, interleukin-6, and vascular endothelial growth factor, stimulating EPCs mobilization and release from bone marrow to peripheral circulation (Patel et al., 2016). After EPCs enter the peripheral blood, they migrate and adhere to the damaged blood vessels, proliferate and differentiate into mature endothelial cells, promote reendothelialization and neovascularization, so as to maintain the integrity of endothelial structure and repair ischemic tissue (Minhajat et al., 2015; Kiewisz et al., 2016). However, it has been shown that Ang II impairs the proliferative and migratory ability of EPCs, resulting in diminished vascular regeneration (Endtmann et al., 2011). Our experimental results also confirmed this point of view. The function of EPCs to repair endothelium and promote angiogenesis was signally inhibited by Ang II, which indicated that EPCs function is impaired in hypertensive population. Rhy improved the damaged cellular function, whereas these effects were abolished by 3-MA.

AMPK is a critical sensor of cellular energy status and a master regulator of metabolism, which plays a key role in energy homeostasis (Garcia and Shaw, 2017). In addition, AMPK regulates growth and coordinates mitochondrial homeostasis (Mihaylova and Shaw, 2011; Herzig and Shaw, 2018). In recent years, AMPK has been found to be closely related to autophagy. AMPK regulates various aspects of the autophagy machinery, and the phosphorylation of AMPK relieves the inhibitory phosphorylation on ULK1 to activate autophagy by reducing mTOR activity (Herzig and Shaw, 2018). Therefore, we detected the activation status of AMPK. Our results showed that Rhy increased the level of p-AMPKα, meaning that Rhy promoted the phosphorylation of AMPK and thus enhanced autophagy. Partial blocking of AMPK pathway after the transfection with siRNA inhibited the promoting effect of Rhy on autophagy. This result also reflected that Rhy regulated autophagy by activating AMPK signaling pathway. A recent study found that Akt2-AMPK double ablation expedites cardiac aging by compromising autophagy and mitophagy (Wang et al., 2019). Therefore, we speculated that the activation of AMPK by Rhy attenuates EPCs senescence by motivating autophagy, but more experiments are needed to verify.

It is necessary to point out that our current study suffers from some limitations. Since we determined the optimal intervention concentration of Ang II; in previous study, other conditions of Ang II were not conducted. Although our study sheds some light on the role of Rhy on autophagy and Ang II-induced EPCs senescence *in vitro*, the results have not been validated in animals or human. In addition, more studies are needed to explore the potential mechanisms of attenuating EPCs senescence.

In summary, our study provided evidence that Ang II induces EPCs senescence and inhibits autophagy, while enhancing autophagy attenuates EPCs senescence. Rhy decelerates EPCs senescence and improves cellular function by rescuing autophagy impaired by Ang II, which is related to the activation of AMPK pathway. EPCs have positive effect in repairing endothelium and improving ischemia, thus, antagonizing EPCs injury, and maintaining the function and number of EPCs can not only effectively repair blood vessels and protect target organs, but also further assist the control of BP by improving the function of endothelium. Our results provide a new insight for the treatment of hypertension. Next, we will further study the effect of Rhy on autophagy and senescence *in vivo* to explore its effect on EPCs damage of hypertension.

## Data Availability Statement

All datasets generated for this study are included in the article/supplementary material.

## Ethics Statement

The experimental protocol used in this study was approved by the Ethics Committee of Shandong University of Traditional Chinese Medicine (approval number: SDUTCM2018629). All methods were performed in accordance with the Guide for the Care and Use of Laboratory Animals (published by the US National Institutes of Health).

## Author Contributions

LL, Y-lL, and CL conceived and designed the study. LL, LZ, X-tL, and J-kJ performed the experiments. LL, LZ, and X-qC analyzed the data. LL wrote the paper. All authors reviewed the manuscript. Each of the authors agrees to be accountable for the content of the work.

## Funding

This work received support by the National Nature Science Foundation of China (81774242), the Major Science and Technology Innovation Project in Shandong Province (2017CXGC1307) and the Special Scientific Research Foundation of National TCM Clinical Research Base (JDZX2015144).

## Conflict of Interest

The authors declare that the research was conducted in the absence of any commercial or financial relationships that could be construed as a potential conflict of interest.

## Abbreviations

EPCs, endothelial progenitor cells; Ang II, angiotensin II; Rhy, rhynchophylline; AMPK, AMP-activated protein kinase; RAAS, renin-angiotensin-aldosterone system; RTA, rhynchophylla total alkaloid; EBM-2, endothelial basal medium-2; Dil-Ac-LDL, 1,1’-dioctadecyl-3,3,3’,3’-tetramethylindocarbocyanine- labeled acetylated low density lipoprotein; FITC-UEA-1, fluorescein isothiocyanate-labeled Ulex europaeus agglutinin 1; SA-β-gal, senescence-associated β-galactosidase; Rap, rapamycin; 3-MA, 3-methyladenine.
